# The alluaudite-type crystal structures of Na_2_(Fe/Co)_2_Co(VO_4_)_3_ and Ag_2_(Fe/Co)_2_Co(VO_4_)_3_

**DOI:** 10.1107/S2056989016009981

**Published:** 2016-06-24

**Authors:** Mohammed Hadouchi, Abderrazzak Assani, Mohamed Saadi, Lahcen El Ammari

**Affiliations:** a Laboratoire de Chimie du Solide Appliquée, Faculty of Sciences, Mohammed V University in Rabat, Avenue Ibn Battouta, BP 1014, Rabat, Morocco

**Keywords:** crystal structure, transition metal vanadates, solid-state reaction synthesis, alluaudite-like structure

## Abstract

Single crystals of the title compounds, disodium di(cobalt/iron) cobalt tris­(orthovanadate), Na_2_(Fe/Co)_2_Co(VO_4_)_3_, and disilver di(cobalt/iron) cobalt tris­(orthovanadate), Ag_2_(Fe/Co)_2_Co(VO_4_)_3_, were grown from a melt consisting of stoichiometric mixtures of three metallic cation precursors and vanadium pentoxide. The difficulty to distinguish between cobalt and iron by using X-ray diffraction alone forced us to explore several models, assuming an oxidation state of +II for Co and +III for Fe and a partial cationic disorder in the Wyckoff site 8*f* containing a mixture of Co and Fe with a statistical distribution for the Na compound and an occupancy ratio of 0.4875:0.5125 (Co:Fe) for the Ag compound. The alluaudite-type structure is made up from [10-1] chains of [(Co,Fe)_2_O_10_] double octa­hedra linked by highly distorted [CoO_6_] octa­hedra *via* a common edge. The chains are linked through VO_4_ tetra­hedra, forming polyhedral sheets perpendicular to [010]. The stacking of the sheets defines two types of channels parallel to [001] where the Na^+^ cations (both with full occupancy) or Ag^+^ cations (one with occupancy 0.97) are located.

## Abstract 2

The transition metal orthovanadates Na_2_(Fe/Co)_2_Co(VO_4_)_3_ and Ag_2_(Fe/Co)_2_Co(VO_4_)_3_ are isotypic and crystallize in an alluaudite-type structure.

## Chemical context   

The needs of the society on the ‘energy front’ is one of the greatest challenges for present and future times. Materials with three-dimensional framework structures delimiting channels, as built of transition metal cations and polyanions (*X*O_4_)^*n*−^, have become a subject of very intensive research worldwide since the discovery of the electrochemical activity of LiFePO_4_ (Padhi *et al.*, 1997*a* ▸,*b* ▸). Hence, new transition metal-based materials adopting open three-dimensional framework structures have been synthesized and investigated by us over the last years. Thereby our attention has focused on the synthesis and characterization of new materials belonging to the family of alluaudites that, according to Moore (1971 ▸), has the general formula *A*(1)*A*(2)*M*(1)*M*(2)_2_(*X*O_4_)_3_. The *A* sites may be occupied by larger mono- and/or divalent cations, while the *M* sites correspond to bi- or trivalent transition metal cations in an octa­hedral environment. Alluaudite-like compounds, having open-framework structures, allow a certain prediction of physical properties and promising practical applications in several fields. For instance, alluaudite-like compounds exhibit electronic and/or ionic conductivity, as has been shown by Warner *et al.* (1993 ▸, 1994 ▸), which make them worthy of investigating their electrochemical performance. Mainly, several alluaudite-like phosphates have been tested as anode and/or cathode materials in Li-ion and/or Na-ion batteries. For example, Li_0.78_Na_0.22_MnPO_4_ was proposed by Kim *et al.* (2014 ▸) as a promising new positive electrode for Li-ion batteries. The sulfates Na_2.44_Mn_1.79_(SO_4_)_3_ (Dwibedi *et al.*, 2015 ▸) and Na_2+2*x*_Fe_2−*x*_(SO_4_)_3_ (Dwibedi *et al.*, 2016 ▸) were tested as electroactive materials for Na-ion batteries. In this context, we have investigated *pseudo*-ternary *A*_2_O/*M*O/P_2_O_5_, *pseudo*-quaternary *A*_2_O/*M*O/Fe_2_O_3_/P_2_O_5_, and more recently, *A*_2_O/*M*O/Fe_2_O_3_/V_2_O_5_ systems synthesized *via* hydro­thermal or solid-state routes, resulting in new alluaudite-like phosphates AgMg_3_(HPO_4_)_2_PO_4_ (Assani *et al.*, 2011 ▸), NaMg_3_(HPO_4_)_2_PO_4_ (Ould Saleck *et al.*, 2015 ▸), Na_2_Co_2_Fe(PO_4_)_3_ (Bouraima *et al.*, 2015 ▸), Na_1.67_Zn_1.67_Fe_1.33_(PO_4_)_3_ (Khmiyas *et al.*, 2015 ▸), and most lately, the first alluaudite-like vanadate (Na_0.70_)(Na_0.70_Mn_0.30_)(Fe^3+^/Fe^2+^)_2_Fe^2+^(VO_4_)_3_ (Benhsina *et al.*, 2016 ▸). As a continuation of our studies of phases with alluaudite-like structures, the present work reports details of the synthesis and crystal structures of the compounds *M*_2_(Fe/Co)_2_Co(VO_4_)_3_ (*M* = Na, Ag).

## Structural commentary   

**Figure 1:**
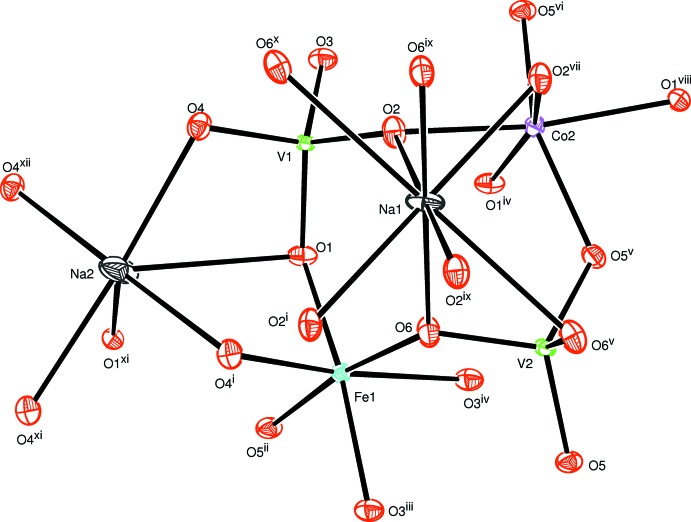
The principal building units in the structure of Na_2_(Fe/Co)_2_Co(VO_4_)_3_. Displacement ellipsoids are drawn at the 50% probability level. [Symmetry codes: (i) *x*, −*y* + 1, *z* + 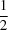; (ii) −*x* + 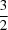, −*y* + 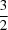, −*z* + 2; (iii) *x*, *y*, *z* + 1; (iv) −*x* + 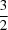, −*y* + 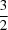, −*z* + 1; (v) −*x* + 1, *y*, −*z* + 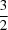; (vi) *x*, *y*, *z* − 1; (vii) −*x* + 1, *y*, −*z* + 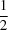; (viii) *x* − 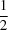, −*y* + 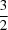, *z* − 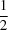; (ix) −*x* + 1, −*y* + 1, −*z* + 1; (*x*) *x*, −*y* + 1, *z* − 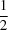; (xi) −*x* + 2, *y*, −*z* + 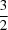; (xii) −*x* + 2, −*y* + 1, −*z* + 1.]

**Figure 3:**
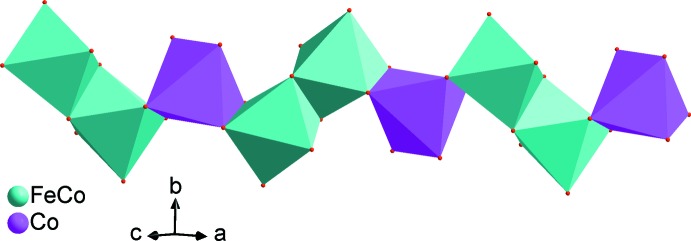
Edge-sharing octa­hedra forming an infinite zigzag chain running along [10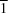]. Data from Na_2_(Fe/Co)_2_Co(VO_4_)_3_.

**Figure 4:**
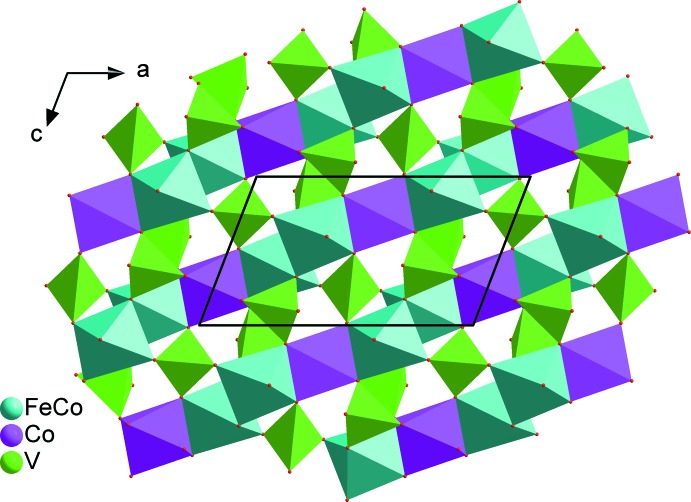
A sheet perpendicular to [010], resulting from the connection of individual chains *via* VO_4_ tetra­hedra. Data from Na_2_(Fe/Co)_2_Co(VO_4_)_3_.

**Figure 5:**
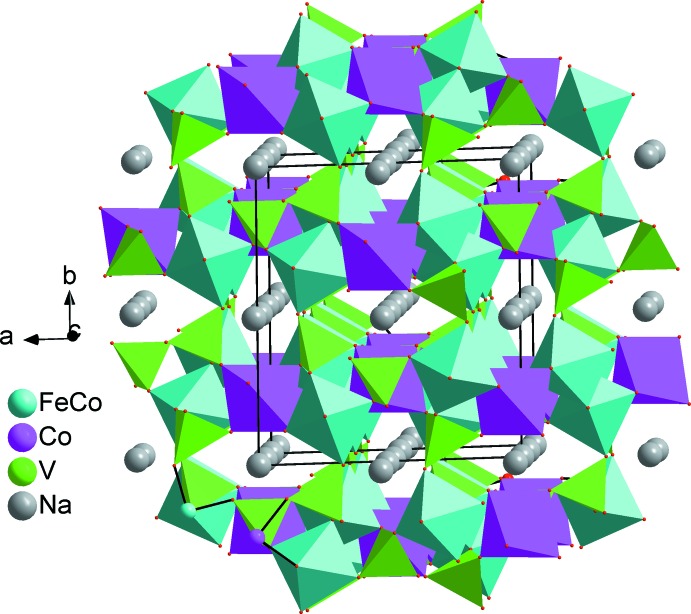
Polyhedral representation of Na_2_(Fe/Co)_2_Co(VO_4_)_3_ showing sodium cations in the channels extending along [001].

The two alluaudite-like vanadates, Na_2_(Fe/Co)_2_Co(VO_4_)_3_ and Ag_2_(Fe/Co)_2_Co(VO_4_)_3_, are isotypic. In the structure of Na_2_(Fe/Co)_2_Co(VO_4_)_3_ all sites are fully occupied and only the cationic site on Wyckoff position 8*f* shows disorder with a statistical distribution of Co and Fe, assuming oxidation state +II for Co and +III for Fe. In the structure of Ag_2_(Fe/Co)_2_Co(VO_4_)_3_ a small deficit in the Ag2 site was considered (occupancy 0.97) that is compensated by a slight excess of Fe (occupancy 0.51) compared with Co (occupancy 0.49) in the 8*f* mixed site, again under the assumption of oxidation state +II for Co and +III for Fe. The (Fe1,Co1) and Co2 sites have octa­hedral environments while the vanadium atoms are located in tetra­hedral environments. The sequence of different polyhedra forming the principal building units are shown in Figs. 1 ▸ and 2 ▸. The mixed-occupied sites containing the (Fe1,Co1) atoms form [(Co,Fe)_2_O_10_] dimers through edge-sharing of a single octa­hedron and are linked by highly distorted [CoO_6_] octa­hedra. The linkage of alternating [CoO_6_] octa­hedra and [(Co,Fe)_2_O_10_] double octa­hedra leads to the formation of infinite chains along the [10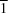] direction (Fig. 3 ▸). The connection of these chains through VO_4_ tetra­hedra makes up sheets perpendicular to [010], as shown in Fig. 4 ▸. The stacking of these sheets defines an open three-dimensional framework delimiting two types of channels parallel to [001] where the *M* ^+^ cations (*M* = Na, Ag) are situated (Fig. 5 ▸). In the sodium compound, the Na1 site is coordinated by eight oxygen atoms with Na1—O distances in the range between 2.4118 (14) and 2.8820 (15) Å, while Na2 is surrounded by six oxygen atoms in a range between 2.4347 (14) and 2.780 (2) Å. In the silver compound, the Ag1 site is coordinated by six oxygen atoms in a range between 2.4244 (12) and 2.5960 (13) Å, whereas the Ag2 site is surrounded by four oxygen atoms in a range between 2.4708 (14) and 2.4779 (14) Å.

## Synthesis and crystallization   

The target compounds were obtained by solid-state reactions. A starting mixture of metallic iron (+ a few drops of HNO_3_), Co(CH_3_COO)_2_·4H_2_O, NH_4_VO_3_ and NaNO_3_ or AgNO_3_ was mixed in molar ratios *M*: Co: Fe: V = 2: 2: 1: 3 (*M* = Na or Ag). The mixture was placed in a platinum crucible and then heated gradually until melting (1253 K). Single crystals were obtained by cooling the molten product to room temperature at rate of 5 Kh^−1^. The resulting mixtures contained pink crystals (for *M* = Na) or green crystals (for *M* = Ag) of a suitable size for the X-ray diffraction study. The powder X-ray diffraction patterns are in good agreement with the simulated patterns, generated from the final structure models of the two compounds (see supplementary material).

## Refinement   

Crystal data, data collection and structure refinement details for both structures are summarized in Table 1 ▸.

As a matter of fact, the distinction between cobalt and iron by X-ray diffraction is nearly impossible. Therefore we have examined several crystallographic models during crystal structure refinements of the title compounds. Based on the stoichiometric ratio of 1:2 for iron and cobalt in the starting materials, we assumed the same ratio in the crystal structures with oxidation states of +II for cobalt and and +III for iron. In the final model, Fe1 and Co1 atoms are constrained to share the same general position 8*f* of the space group *C*2/*c*. Electrical neutrality and bond valence sum calculations of all atoms (Brown & Altermatt, 1985 ▸) in the structures are in reasonable agreement with the final models. Bond valence sums (in valence units) for Na_2_(Fe/Co)_2_Co(VO_4_)_3_ are 1.07 for Na1, 0.86 for Na2, 2.24 for Co1, 1.97 for Co2, 2.69 for Fe1, 5.01 for V1, and 4.99 for V2. Values of the bond valence sums calculated for all oxygen atoms are between 1.90 and 2.07. Bond valence sums for Ag_2_(Fe/Co)_2_Co(VO_4_)_3_ are 1.01 for Ag1, O.72 for Ag2, 2.27 for Co1, 1.98 for Co2, 2.72 for Fe1, 4.99 for V1, and 4.94 for V2. The values of the O atoms are in the range 1.94 to 2.03. A very similar cationic distribution was observed by Yakubovich *et al.* (1977 ▸) in the alluaudite-type phosphate Na_2_(Fe^3+^/Fe^2+^)_2_Fe^2+^(PO_4_)_3_.

Refinement of Na_2_(Fe/Co)_2_Co(VO_4_)_3_: Co1 and Fe1 were constrained to share the same site in a statistical occupation with common displacement parameters. Reflection (132) probably was affected by the beam-stop and was omitted from the refinement. The remaining electron densities (max/min) in the final Fourier map were 0.46 Å and 0.71 Å away from atoms Na1 and Na2, respectively.

Refinement of Ag_2_(Fe/Co)_2_Co(VO_4_)_3_: The coordinates and displacement factors of Co1 and Fe1 atoms were refined independent from each other. An underoccupation of the Ag2 site was modelled with an occupancy of 0.97 which made it necessary to constrain the occupancies of the Co1 site (0.4875) and Fe1 site (0.5125) for electroneutrality. The remaining electron densities (max/min) in the final Fourier map were 0.61 Å and 0.66 Å away from Ag2.

## Supplementary Material

Crystal structure: contains datablock(s) I, II, global. DOI: 10.1107/S2056989016009981/wm5292sup1.cif

Structure factors: contains datablock(s) I. DOI: 10.1107/S2056989016009981/wm5292Isup2.hkl

Structure factors: contains datablock(s) II. DOI: 10.1107/S2056989016009981/wm5292IIsup3.hkl

CCDC references: 1486597, 1486596

Additional supporting information:  crystallographic information; 3D view; checkCIF report

## Figures and Tables

**Figure 2:**
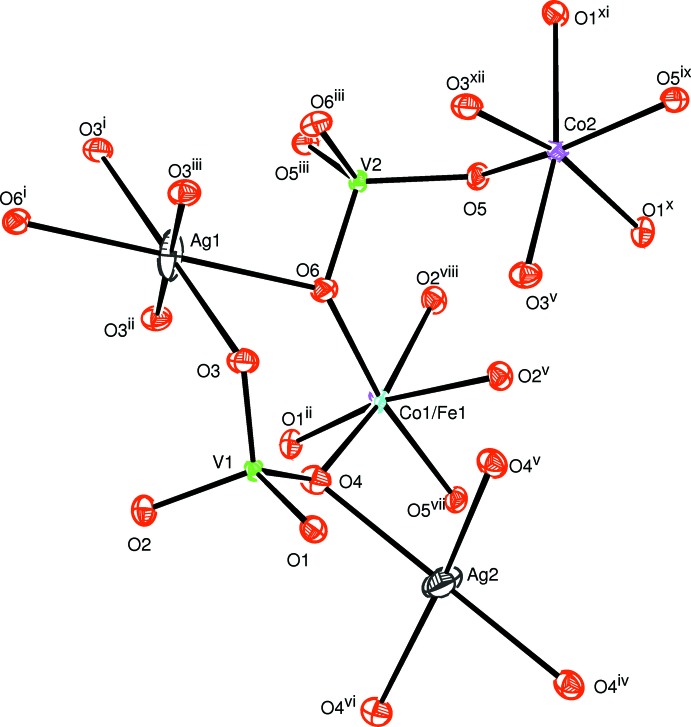
The principal building units in the structure of Ag_2_(Fe/Co)_2_Co(VO_4_)_3_. Displacement ellipsoids are drawn at the 50% probability level. [Symmetry codes: (i) *x*, −*y* + 1, *z* + 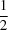; (ii) −*x* + 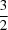, −*y* + 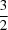, −*z* + 2; (iii) *x*, *y*, *z* + 1; (iv) −*x* + 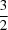, −*y* + 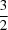, −*z* + 1; (v) −*x* + 1, *y*, −*z* + 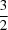; (vi) *x*, *y*, *z* − 1; (vii) −*x* + 1, *y*, −*z* + 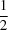; (viii) *x* − 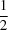, −*y* + 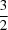, *z* − 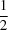; (ix) −*x* + 1, −*y* + 1, −*z* + 1; (*x*) *x*, −*y* + 1, *z* − 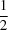; (xi) −*x* + 2, *y*, −*z* + 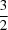; (xii) −*x* + 2, −*y* + 1, −*z* + 1.]

**Table 1:** Experimental details

	(I)	(II)
Crystal data		
Chemical formula	Na_2_(Co_0.5_Fe_0.5_)_2_Co(VO_4_)_3_	Ag_1.97_(Co_0.49_Fe_0.51_)_2_Co(VO_4_)_3_
*M*_r_	564.51	730.96
Crystal system, space group	Monoclinic, *C*2/*c*	Monoclinic, *C*2/*c*
Temperature (K)	296	296
*a*, *b*, *c* (Å)	11.7258 (2), 12.7819 (2), 6.8264 (1)	11.7846 (4), 12.8314 (4), 6.8064 (2)
β (°)	111.069 (1)	111.001 (1)
*V* (Å^3^)	954.73 (3)	960.85 (5)
*Z*	4	4
Radiation type	Mo *K*α	Mo *K*α
μ (mm^−1^)	7.85	11.60
Crystal size (mm)	0.32 × 0.25 × 0.19	0.34 × 0.22 × 0.17

Data collection		
Diffractometer	Bruker X8 APEX	Bruker X8 APEX
Absorption correction	Multi-scan (*SADABS*; Bruker, 2009 ▸)	Multi-scan (*SADABS*; Bruker, 2009 ▸)
*T*_min_, *T*_max_	0.572, 0.747	0.439, 0.747
No. of measured, independent and observed [*I* > 2σ(*I*)] reflections	17675, 2094, 1893	15366, 2113, 1987
*R*_int_	0.047	0.039
(sin θ/λ)_max_ (Å^−1^)	0.806	0.806

Refinement		
*R*[*F* ^2^ > 2σ(*F* ^2^)], *wR*(*F* ^2^), *S*	0.022, 0.054, 1.10	0.018, 0.041, 1.14
No. of reflections	2094	2113
No. of parameters	95	104
Δρ_max_, Δρ_min_ (e Å^−3^)	0.88, −1.00	0.80, −1.66

Computer programs: *APEX2* and *SAINT* (Bruker, 2009 ▸), *SHELXT2014* (Sheldrick, 2015*a* ▸), *SHELXL2014* (Sheldrick, 2015*b* ▸), *ORTEP-3 for Windows* (Farrugia, 2012 ▸), *DIAMOND* (Brandenburg, 2006 ▸) and *publCIF* (Westrip, 2010 ▸).

## supplementary crystallographic information

### (I) Disodium di(cobalt/iron) cobalt tris(orthovanadate) Crystal data

**Table:**

Na_2_(Co·Fe)_2_Co(VO_4_)_3_	*F*(000) = 1068
*M**_r_* = 564.51	*D*_x_ = 3.927 Mg m^−^^3^
Monoclinic, *C*2/*c*	Mo *K*α radiation, λ = 0.71073 Å
*a* = 11.7258 (2) Å	Cell parameters from 2094 reflections
*b* = 12.7819 (2) Å	θ = 2.5–35.0°
*c* = 6.8264 (1) Å	µ = 7.85 mm^−^^1^
β = 111.069 (1)°	*T* = 296 K
*V* = 954.73 (3) Å^3^	Block, pink
*Z* = 4	0.32 × 0.25 × 0.19 mm

### (I) Disodium di(cobalt/iron) cobalt tris(orthovanadate) Data collection

**Table:**

Bruker X8 APEX diffractometer	2094 independent reflections
Radiation source: fine-focus sealed tube	1893 reflections with *I* > 2σ(*I*)
Graphite monochromator	*R*_int_ = 0.047
φ and ω scans	θ_max_ = 35.0°, θ_min_ = 2.5°
Absorption correction: multi-scan (*SADABS*; Bruker, 2009)	*h* = −17→18
*T*_min_ = 0.572, *T*_max_ = 0.747	*k* = −20→20
17675 measured reflections	*l* = −10→10

### (I) Disodium di(cobalt/iron) cobalt tris(orthovanadate) Refinement

**Table:**

Refinement on *F*^2^	95 parameters
Least-squares matrix: full	0 restraints
*R*[*F*^2^ > 2σ(*F*^2^)] = 0.022	*w* = 1/[σ^2^(*F*_o_^2^) + (0.0186*P*)^2^ + 2.754*P*] where *P* = (*F*_o_^2^ + 2*F*_c_^2^)/3
*wR*(*F*^2^) = 0.054	(Δ/σ)_max_ = 0.001
*S* = 1.10	Δρ_max_ = 0.88 e Å^−^^3^
2094 reflections	Δρ_min_ = −1.00 e Å^−^^3^

### (I) Disodium di(cobalt/iron) cobalt tris(orthovanadate) Special details

**Table:**

Geometry. All esds (except the esd in the dihedral angle between two l.s. planes) are estimated using the full covariance matrix. The cell esds are taken into account individually in the estimation of esds in distances, angles and torsion angles; correlations between esds in cell parameters are only used when they are defined by crystal symmetry. An approximate (isotropic) treatment of cell esds is used for estimating esds involving l.s. planes.

### (I) Disodium di(cobalt/iron) cobalt tris(orthovanadate) Fractional atomic coordinates and isotropic or equivalent isotropic displacement parameters (Å^2^)

**Table:**

	*x*	*y*	*z*	*U*_iso_*/*U*_eq_	Occ. (<1)
Fe1	0.79142 (2)	0.66131 (2)	0.88043 (4)	0.00595 (6)	0.5
Co1	0.79142 (2)	0.66131 (2)	0.88043 (4)	0.00595 (6)	0.5
Co2	0.5000	0.73184 (3)	0.2500	0.00727 (7)	
V1	0.77138 (3)	0.61298 (2)	0.38340 (4)	0.00555 (6)	
V2	0.5000	0.70722 (3)	0.7500	0.00629 (8)	
Na1	0.5000	0.5000	0.5000	0.0123 (2)	
Na2	1.0000	0.50245 (14)	0.7500	0.0257 (3)	
O1	0.84286 (13)	0.67273 (11)	0.6264 (2)	0.0102 (2)	
O2	0.62045 (12)	0.60248 (11)	0.3329 (2)	0.0116 (2)	
O3	0.78619 (13)	0.68309 (11)	0.1758 (2)	0.0113 (2)	
O6	0.61413 (13)	0.62261 (11)	0.7671 (2)	0.0120 (2)	
O5	0.53637 (12)	0.77735 (11)	0.9853 (2)	0.0104 (2)	
O4	0.84074 (13)	0.49296 (11)	0.4032 (2)	0.0108 (2)	

### (I) Disodium di(cobalt/iron) cobalt tris(orthovanadate) Atomic displacement parameters (Å^2^)

**Table:**

	*U*^11^	*U*^22^	*U*^33^	*U*^12^	*U*^13^	*U*^23^
Fe1	0.00562 (10)	0.00715 (11)	0.00541 (10)	−0.00056 (7)	0.00237 (7)	−0.00051 (7)
Co1	0.00562 (10)	0.00715 (11)	0.00541 (10)	−0.00056 (7)	0.00237 (7)	−0.00051 (7)
Co2	0.00669 (14)	0.00821 (15)	0.00752 (13)	0.000	0.00330 (11)	0.000
V1	0.00559 (12)	0.00635 (12)	0.00449 (11)	−0.00001 (9)	0.00155 (9)	−0.00011 (8)
V2	0.00700 (17)	0.00647 (17)	0.00459 (15)	0.000	0.00113 (12)	0.000
Na1	0.0208 (6)	0.0070 (5)	0.0074 (4)	−0.0057 (4)	0.0030 (4)	−0.0015 (3)
Na2	0.0097 (5)	0.0565 (10)	0.0105 (5)	0.000	0.0031 (4)	0.000
O1	0.0115 (6)	0.0116 (6)	0.0074 (5)	−0.0037 (5)	0.0031 (4)	−0.0016 (4)
O2	0.0077 (5)	0.0107 (6)	0.0158 (6)	−0.0008 (4)	0.0035 (5)	0.0004 (5)
O3	0.0138 (6)	0.0126 (6)	0.0080 (5)	−0.0004 (5)	0.0043 (4)	0.0006 (4)
O6	0.0092 (6)	0.0098 (6)	0.0148 (6)	0.0012 (4)	0.0015 (5)	−0.0019 (5)
O5	0.0075 (5)	0.0156 (6)	0.0082 (5)	−0.0017 (5)	0.0028 (4)	−0.0025 (4)
O4	0.0102 (6)	0.0092 (6)	0.0129 (6)	0.0002 (4)	0.0042 (5)	−0.0015 (4)

### (I) Disodium di(cobalt/iron) cobalt tris(orthovanadate) Geometric parameters (Å, º)

**Table:**

Fe1—O6	2.0021 (14)	V2—O6^v^	1.6917 (14)
Fe1—O1	2.0359 (14)	V2—O5	1.7531 (13)
Fe1—O4^i^	2.0451 (14)	V2—O5^v^	1.7531 (13)
Fe1—O5^ii^	2.0497 (14)	Na1—O6	2.4118 (14)
Fe1—O3^iii^	2.0581 (14)	Na1—O6^ix^	2.4119 (14)
Fe1—O3^iv^	2.1633 (14)	Na1—O2^ix^	2.4841 (14)
Co2—O5^v^	2.0831 (14)	Na1—O2	2.4841 (14)
Co2—O5^vi^	2.0831 (14)	Na1—O2^i^	2.5626 (14)
Co2—O2	2.1153 (14)	Na1—O2^vii^	2.5626 (14)
Co2—O2^vii^	2.1153 (14)	Na1—O6^x^	2.8820 (15)
Co2—O1^iv^	2.1159 (13)	Na1—O6^v^	2.8820 (15)
Co2—O1^viii^	2.1159 (13)	Na2—O4^xi^	2.4347 (14)
V1—O2	1.6823 (14)	Na2—O4	2.4347 (14)
V1—O4	1.7188 (14)	Na2—O4^i^	2.4472 (15)
V1—O3	1.7374 (14)	Na2—O4^xii^	2.4472 (15)
V1—O1	1.7428 (13)	Na2—O1	2.780 (2)
V2—O6	1.6917 (14)	Na2—O1^xi^	2.780 (2)
			
O6—Fe1—O1	105.84 (6)	O6—Na1—O2^ix^	104.36 (5)
O6—Fe1—O4^i^	90.98 (6)	O6^ix^—Na1—O2^ix^	75.63 (5)
O1—Fe1—O4^i^	88.38 (6)	O6—Na1—O2	75.64 (5)
O6—Fe1—O5^ii^	170.54 (6)	O6^ix^—Na1—O2	104.37 (5)
O1—Fe1—O5^ii^	78.91 (5)	O2^ix^—Na1—O2	180.0
O4^i^—Fe1—O5^ii^	97.39 (6)	O6—Na1—O2^i^	71.50 (5)
O6—Fe1—O3^iii^	91.12 (6)	O6^ix^—Na1—O2^i^	108.50 (5)
O1—Fe1—O3^iii^	161.29 (6)	O2^ix^—Na1—O2^i^	63.02 (6)
O4^i^—Fe1—O3^iii^	99.39 (6)	O2—Na1—O2^i^	116.98 (6)
O5^ii^—Fe1—O3^iii^	83.21 (5)	O6—Na1—O2^vii^	108.50 (5)
O6—Fe1—O3^iv^	81.17 (6)	O6^ix^—Na1—O2^vii^	71.50 (5)
O1—Fe1—O3^iv^	91.00 (5)	O2^ix^—Na1—O2^vii^	116.98 (6)
O4^i^—Fe1—O3^iv^	171.64 (6)	O2—Na1—O2^vii^	63.02 (6)
O5^ii^—Fe1—O3^iv^	90.67 (6)	O2^i^—Na1—O2^vii^	180.0
O3^iii^—Fe1—O3^iv^	83.72 (5)	O6—Na1—O6^x^	121.93 (6)
O5^v^—Co2—O5^vi^	147.57 (8)	O6^ix^—Na1—O6^x^	58.07 (6)
O5^v^—Co2—O2	108.15 (5)	O2^ix^—Na1—O6^x^	114.84 (4)
O5^vi^—Co2—O2	97.18 (6)	O2—Na1—O6^x^	65.16 (4)
O5^v^—Co2—O2^vii^	97.18 (6)	O2^i^—Na1—O6^x^	89.66 (4)
O5^vi^—Co2—O2^vii^	108.15 (5)	O2^vii^—Na1—O6^x^	90.34 (4)
O2—Co2—O2^vii^	77.17 (8)	O6—Na1—O6^v^	58.07 (6)
O5^v^—Co2—O1^iv^	85.04 (5)	O6^ix^—Na1—O6^v^	121.93 (6)
O5^vi^—Co2—O1^iv^	76.38 (5)	O2^ix^—Na1—O6^v^	65.16 (4)
O2—Co2—O1^iv^	86.67 (5)	O2—Na1—O6^v^	114.84 (4)
O2^vii^—Co2—O1^iv^	163.59 (6)	O2^i^—Na1—O6^v^	90.34 (4)
O5^v^—Co2—O1^viii^	76.38 (5)	O2^vii^—Na1—O6^v^	89.66 (4)
O5^vi^—Co2—O1^viii^	85.04 (5)	O6^x^—Na1—O6^v^	180.0
O2—Co2—O1^viii^	163.59 (6)	O4^xi^—Na2—O4	174.29 (11)
O2^vii^—Co2—O1^viii^	86.67 (5)	O4^xi^—Na2—O4^i^	91.26 (5)
O1^iv^—Co2—O1^viii^	109.60 (8)	O4—Na2—O4^i^	88.87 (5)
O2—V1—O4	112.22 (7)	O4^xi^—Na2—O4^xii^	88.87 (5)
O2—V1—O3	106.27 (7)	O4—Na2—O4^xii^	91.26 (5)
O4—V1—O3	109.96 (7)	O4^i^—Na2—O4^xii^	177.25 (11)
O2—V1—O1	109.92 (7)	O4^xi^—Na2—O1	121.82 (7)
O4—V1—O1	105.32 (7)	O4—Na2—O1	63.31 (5)
O3—V1—O1	113.29 (7)	O4^i^—Na2—O1	65.58 (5)
O6—V2—O6^v^	100.53 (10)	O4^xii^—Na2—O1	112.08 (6)
O6—V2—O5	109.75 (7)	O4^xi^—Na2—O1^xi^	63.31 (5)
O6^v^—V2—O5	108.41 (7)	O4—Na2—O1^xi^	121.82 (7)
O6—V2—O5^v^	108.42 (7)	O4^i^—Na2—O1^xi^	112.08 (6)
O6^v^—V2—O5^v^	109.75 (7)	O4^xii^—Na2—O1^xi^	65.58 (5)
O5—V2—O5^v^	118.49 (10)	O1—Na2—O1^xi^	76.92 (7)
O6—Na1—O6^ix^	180.0		

Symmetry codes: (i) *x*, −*y*+1, *z*+1/2; (ii) −*x*+3/2, −*y*+3/2, −*z*+2; (iii) *x*, *y*, *z*+1; (iv) −*x*+3/2, −*y*+3/2, −*z*+1; (v) −*x*+1, *y*, −*z*+3/2; (vi) *x*, *y*, *z*−1; (vii) −*x*+1, *y*, −*z*+1/2; (viii) *x*−1/2, −*y*+3/2, *z*−1/2; (ix) −*x*+1, −*y*+1, −*z*+1; (x) *x*, −*y*+1, *z*−1/2; (xi) −*x*+2, *y*, −*z*+3/2; (xii) −*x*+2, −*y*+1, −*z*+1.

### (II) Disilver di(cobalt/iron) cobalt tris(orthovanadate) Crystal data

**Table:**

Ag_1.97_(Co_0.49_Fe_0.51_)_2_Co(VO_4_)_3_	*F*(000) = 1350
*M**_r_* = 730.96	*D*_x_ = 5.053 Mg m^−^^3^
Monoclinic, *C*2/*c*	Mo *K*α radiation, λ = 0.71073 Å
*a* = 11.7846 (4) Å	Cell parameters from 2113 reflections
*b* = 12.8314 (4) Å	θ = 2.4–35.0°
*c* = 6.8064 (2) Å	µ = 11.60 mm^−^^1^
β = 111.001 (1)°	*T* = 296 K
*V* = 960.85 (5) Å^3^	Block, green
*Z* = 4	0.34 × 0.22 × 0.17 mm

### (II) Disilver di(cobalt/iron) cobalt tris(orthovanadate) Data collection

**Table:**

Bruker X8 APEX diffractometer	2113 independent reflections
Radiation source: fine-focus sealed tube	1987 reflections with *I* > 2σ(*I*)
Graphite monochromator	*R*_int_ = 0.039
φ and ω scans	θ_max_ = 35.0°, θ_min_ = 2.4°
Absorption correction: multi-scan (*SADABS*; Bruker, 2009)	*h* = −18→18
*T*_min_ = 0.439, *T*_max_ = 0.747	*k* = −20→20
15366 measured reflections	*l* = −9→10

### (II) Disilver di(cobalt/iron) cobalt tris(orthovanadate) Refinement

**Table:**

Refinement on *F*^2^	0 restraints
Least-squares matrix: full	*w* = 1/[σ^2^(*F*_o_^2^) + (0.0093*P*)^2^ + 2.3975*P*] where *P* = (*F*_o_^2^ + 2*F*_c_^2^)/3
*R*[*F*^2^ > 2σ(*F*^2^)] = 0.018	(Δ/σ)_max_ = 0.001
*wR*(*F*^2^) = 0.041	Δρ_max_ = 0.80 e Å^−^^3^
*S* = 1.14	Δρ_min_ = −1.66 e Å^−^^3^
2113 reflections	Extinction correction: SHELXL2014 (Sheldrick, 2015b), Fc^*^=kFc[1+0.001xFc^2^λ^3^/sin(2θ)]^-1/4^
104 parameters	Extinction coefficient: 0.00190 (7)

### (II) Disilver di(cobalt/iron) cobalt tris(orthovanadate) Special details

**Table:**

Geometry. All esds (except the esd in the dihedral angle between two l.s. planes) are estimated using the full covariance matrix. The cell esds are taken into account individually in the estimation of esds in distances, angles and torsion angles; correlations between esds in cell parameters are only used when they are defined by crystal symmetry. An approximate (isotropic) treatment of cell esds is used for estimating esds involving l.s. planes.

### (II) Disilver di(cobalt/iron) cobalt tris(orthovanadate) Fractional atomic coordinates and isotropic or equivalent isotropic displacement parameters (Å^2^)

**Table:**

	*x*	*y*	*z*	*U*_iso_*/*U*_eq_	Occ. (<1)
Ag1	0.0000	0.5000	0.0000	0.02187 (6)	
Ag2	0.5000	0.50840 (2)	0.7500	0.02578 (6)	0.97
Co1	0.2919 (4)	0.6616 (4)	0.3791 (8)	0.0066 (12)	0.4875
Fe1	0.2923 (4)	0.6620 (4)	0.3814 (8)	0.0061 (12)	0.5125
Co2	0.0000	0.73364 (2)	0.7500	0.00755 (6)	
V1	0.27106 (2)	0.38672 (2)	0.38255 (4)	0.00573 (5)	
V2	0.0000	0.70581 (3)	0.2500	0.00609 (6)	
O1	0.34160 (11)	0.32595 (9)	0.62391 (19)	0.0103 (2)	
O2	0.28472 (11)	0.31626 (10)	0.17435 (19)	0.0109 (2)	
O3	0.12124 (11)	0.39463 (10)	0.3352 (2)	0.0121 (2)	
O4	0.33731 (12)	0.50845 (9)	0.3988 (2)	0.0123 (2)	
O5	0.03699 (11)	0.77654 (10)	0.48520 (19)	0.0100 (2)	
O6	0.11558 (11)	0.62338 (9)	0.2663 (2)	0.0114 (2)	

### (II) Disilver di(cobalt/iron) cobalt tris(orthovanadate) Atomic displacement parameters (Å^2^)

**Table:**

	*U*^11^	*U*^22^	*U*^33^	*U*^12^	*U*^13^	*U*^23^
Ag1	0.03724 (13)	0.01563 (9)	0.01248 (9)	−0.01172 (8)	0.00860 (8)	−0.00330 (6)
Ag2	0.01181 (9)	0.04845 (15)	0.01618 (10)	0.000	0.00390 (8)	0.000
Co1	0.0072 (18)	0.0061 (17)	0.0085 (18)	0.0017 (12)	0.0052 (12)	−0.0004 (12)
Fe1	0.0059 (17)	0.0082 (18)	0.0032 (15)	−0.0027 (12)	0.0007 (11)	−0.0007 (11)
Co2	0.00710 (12)	0.00870 (12)	0.00779 (13)	0.000	0.00380 (10)	0.000
V1	0.00651 (10)	0.00586 (10)	0.00488 (10)	0.00018 (7)	0.00212 (8)	0.00004 (7)
V2	0.00679 (14)	0.00648 (13)	0.00465 (14)	0.000	0.00161 (11)	0.000
O1	0.0119 (5)	0.0111 (5)	0.0081 (5)	0.0027 (4)	0.0038 (4)	0.0013 (4)
O2	0.0129 (5)	0.0121 (5)	0.0083 (5)	−0.0007 (4)	0.0045 (4)	−0.0007 (4)
O3	0.0094 (5)	0.0113 (5)	0.0156 (6)	0.0007 (4)	0.0045 (4)	−0.0005 (4)
O4	0.0132 (5)	0.0087 (5)	0.0156 (6)	−0.0002 (4)	0.0060 (5)	0.0010 (4)
O5	0.0091 (5)	0.0136 (5)	0.0078 (5)	−0.0018 (4)	0.0036 (4)	−0.0019 (4)
O6	0.0088 (5)	0.0103 (5)	0.0137 (5)	0.0000 (4)	0.0024 (4)	−0.0021 (4)

### (II) Disilver di(cobalt/iron) cobalt tris(orthovanadate) Geometric parameters (Å, º)

**Table:**

Ag1—O6^i^	2.4244 (12)	Fe1—O1^ii^	2.040 (6)
Ag1—O6	2.4244 (12)	Fe1—O5^vii^	2.045 (5)
Ag1—O3^ii^	2.5051 (13)	Fe1—O2^v^	2.047 (5)
Ag1—O3^iii^	2.5051 (13)	Fe1—O2^viii^	2.154 (5)
Ag1—O3	2.5960 (13)	Co2—O5^ix^	2.0747 (12)
Ag1—O3^i^	2.5960 (13)	Co2—O5	2.0748 (12)
Ag2—O4	2.4708 (14)	Co2—O1^x^	2.1156 (12)
Ag2—O4^iv^	2.4709 (14)	Co2—O1^xi^	2.1156 (12)
Ag2—O4^v^	2.4779 (14)	Co2—O3^xii^	2.1197 (13)
Ag2—O4^vi^	2.4779 (14)	Co2—O3^v^	2.1197 (13)
Co1—O6	2.001 (5)	V1—O3	1.6804 (13)
Co1—O1^ii^	2.028 (6)	V1—O4	1.7319 (12)
Co1—O4	2.028 (5)	V1—O2	1.7363 (12)
Co1—O5^vii^	2.053 (5)	V1—O1	1.7375 (12)
Co1—O2^v^	2.061 (5)	V2—O6	1.6965 (12)
Co1—O2^viii^	2.157 (5)	V2—O6^iii^	1.6965 (12)
Fe1—O6	2.007 (5)	V2—O5^iii^	1.7539 (12)
Fe1—O4	2.033 (5)	V2—O5	1.7539 (12)
			
O6^i^—Ag1—O6	180.0	O6—Fe1—O5^vii^	170.5 (3)
O6^i^—Ag1—O3^ii^	105.99 (4)	O4—Fe1—O5^vii^	98.8 (2)
O6—Ag1—O3^ii^	74.01 (4)	O1^ii^—Fe1—O5^vii^	79.3 (2)
O6^i^—Ag1—O3^iii^	74.01 (4)	O6—Fe1—O2^v^	90.7 (2)
O6—Ag1—O3^iii^	105.99 (4)	O4—Fe1—O2^v^	100.2 (2)
O3^ii^—Ag1—O3^iii^	180.0	O1^ii^—Fe1—O2^v^	162.1 (3)
O6^i^—Ag1—O3	107.57 (4)	O5^vii^—Fe1—O2^v^	83.95 (17)
O6—Ag1—O3	72.43 (4)	O6—Fe1—O2^viii^	81.09 (17)
O3^ii^—Ag1—O3	116.87 (5)	O4—Fe1—O2^viii^	170.3 (2)
O3^iii^—Ag1—O3	63.13 (5)	O1^ii^—Fe1—O2^viii^	90.6 (2)
O6^i^—Ag1—O3^i^	72.43 (4)	O5^vii^—Fe1—O2^viii^	90.5 (2)
O6—Ag1—O3^i^	107.57 (4)	O2^v^—Fe1—O2^viii^	83.31 (18)
O3^ii^—Ag1—O3^i^	63.13 (5)	O5^ix^—Co2—O5	149.23 (7)
O3^iii^—Ag1—O3^i^	116.87 (5)	O5^ix^—Co2—O1^x^	85.91 (5)
O3—Ag1—O3^i^	180.0	O5—Co2—O1^x^	76.96 (5)
O4—Ag2—O4^iv^	179.97 (6)	O5^ix^—Co2—O1^xi^	76.96 (5)
O4—Ag2—O4^v^	87.12 (4)	O5—Co2—O1^xi^	85.91 (5)
O4^iv^—Ag2—O4^v^	92.89 (4)	O1^x^—Co2—O1^xi^	111.91 (7)
O4—Ag2—O4^vi^	92.89 (4)	O5^ix^—Co2—O3^xii^	96.48 (5)
O4^iv^—Ag2—O4^vi^	87.12 (4)	O5—Co2—O3^xii^	107.40 (5)
O4^v^—Ag2—O4^vi^	169.99 (6)	O1^x^—Co2—O3^xii^	162.94 (5)
O6—Co1—O1^ii^	105.6 (2)	O1^xi^—Co2—O3^xii^	85.03 (5)
O6—Co1—O4	90.1 (2)	O5^ix^—Co2—O3^v^	107.40 (5)
O1^ii^—Co1—O4	89.02 (19)	O5—Co2—O3^v^	96.48 (5)
O6—Co1—O5^vii^	170.0 (3)	O1^x^—Co2—O3^v^	85.03 (5)
O1^ii^—Co1—O5^vii^	79.4 (2)	O1^xi^—Co2—O3^v^	162.94 (5)
O4—Co1—O5^vii^	98.73 (19)	O3^xii^—Co2—O3^v^	78.12 (7)
O6—Co1—O2^v^	90.4 (2)	O3—V1—O4	112.11 (6)
O1^ii^—Co1—O2^v^	161.7 (3)	O3—V1—O2	105.86 (6)
O4—Co1—O2^v^	99.9 (2)	O4—V1—O2	110.50 (6)
O5^vii^—Co1—O2^v^	83.40 (18)	O3—V1—O1	108.79 (6)
O6—Co1—O2^viii^	81.15 (16)	O4—V1—O1	107.01 (6)
O1^ii^—Co1—O2^viii^	90.9 (2)	O2—V1—O1	112.65 (6)
O4—Co1—O2^viii^	170.8 (3)	O6—V2—O6^iii^	102.86 (8)
O5^vii^—Co1—O2^viii^	90.3 (2)	O6—V2—O5^iii^	108.27 (6)
O2^v^—Co1—O2^viii^	82.90 (17)	O6^iii^—V2—O5^iii^	109.38 (6)
O6—Fe1—O4	89.8 (2)	O6—V2—O5	109.37 (6)
O6—Fe1—O1^ii^	105.0 (2)	O6^iii^—V2—O5	108.27 (6)
O4—Fe1—O1^ii^	88.6 (2)	O5^iii^—V2—O5	117.68 (8)

Symmetry codes: (i) −*x*, −*y*+1, −*z*; (ii) *x*, −*y*+1, *z*−1/2; (iii) −*x*, *y*, −*z*+1/2; (iv) −*x*+1, *y*, −*z*+3/2; (v) *x*, −*y*+1, *z*+1/2; (vi) −*x*+1, −*y*+1, −*z*+1; (vii) −*x*+1/2, −*y*+3/2, −*z*+1; (viii) −*x*+1/2, *y*+1/2, −*z*+1/2; (ix) −*x*, *y*, −*z*+3/2; (x) −*x*+1/2, *y*+1/2, −*z*+3/2; (xi) *x*−1/2, *y*+1/2, *z*; (xii) −*x*, −*y*+1, −*z*+1.
